# The dual association of serum uric acid with the functional outcomes of patients after hip arthroplasty: 1-year follow-up study

**DOI:** 10.3389/fsurg.2025.1661819

**Published:** 2025-09-26

**Authors:** Ming Xia, Yu Han, Lihui Sun, Dongbo Li, Chunquan Zhu, Dongsong Li

**Affiliations:** Department of Orthopedics, The First Hospital of Jilin University, Changchun, Jilin, China

**Keywords:** uric acid, hip arthroplasty, functional outcomes, nonlinear association, oxidative stress

## Abstract

**Background:**

Serum uric acid (UA) exhibits dual biological roles as both an antioxidant and a pro-oxidant, yet its impact on functional recovery after hip arthroplasty remains unclear. This study investigated the nonlinear relationship between UA levels and 1-year postoperative outcomes in patients undergoing hip arthroplasty.

**Methods:**

In this single-center observational study, 468 hip arthroplasty patients (September 2018–September 2023) were stratified into functional independence (FIM ≥108) and non-independence groups. Serum UA was categorized as low, middle, or high. Functional outcomes were assessed using the UCLA Activity Scale (UCLAAS) and Patient-Reported Satisfaction (PRS) metrics. Restricted cubic splines (RCS) and multivariable regression models evaluated nonlinear and linear associations, adjusted for age, comorbidities, and laboratory parameters.

**Results:**

A U-shaped relationship emerged between UA levels and functional independence (*p* < 0.01 for nonlinearity). Both low UA (OR = 2.09, 95% CI:1.14–3.85) and high UA (OR = 3.74, 95% CI:1.89–7.41) independently predicted reduced functional independence. Secondary outcomes exhibited domain-specific effects: only high UA correlated with poorer mobility (UCLAAS: *β* = −0.53, *p* = 0.015). Multivariable adjustments confirmed the robustness of these associations.

**Conclusion:**

Serum UA demonstrates a dual, nonlinear association with functional recovery after hip arthroplasty, where extremes perturb redox balance and bone remodeling. Monitoring perioperative UA levels and targeting individualized thresholds may optimize rehabilitation strategies.

## Introduction

Hip arthroplasty, also known as hip replacement surgery, is a widely performed procedure. Arthroplasty aims to alleviate pain and restore mobility in patients with severe hip joint damage, typically caused by osteoarthritis, fractures, or other degenerative conditions (1). Despite significant advancements in surgical techniques and postoperative care, functional recovery and long-term outcomes remain variable among patients (2). Identifying prognostic factors influencing recovery is essential for optimizing patient management and improving postoperative quality of life (3). Among these factors, serum uric acid (UA) has emerged as a potential biomarker with a dual role in postoperative outcomes (4).

Serum uric acid (UA) is the final product of purine metabolism and plays a complex role in human health (5). At physiological levels, UA acts as a potent antioxidant, neutralizing free radicals and protecting cells from oxidative damage (6). However, hyperuricemia (HUA) has been proven to be associated with adverse health outcomes. Hyperuricemia is linked to gout, cardiovascular diseases, and chronic kidney disease, while hypouricemia may exacerbate oxidative stress and impair cellular function (7). Recent studies have explored the paradoxical relationship between UA and bone health, suggesting that UA may influence bone metabolism and fracture healing (8–10).

The dual effects of UA on bone and functional outcomes may be mediated by its complex interactions with oxidative stress, inflammation, and bone remodeling processes (11, 12). Moderate UA levels appear to promote bone health by enhancing antioxidant defenses and supporting bone repair (13). In contrast, excessive UA can trigger inflammatory responses and endothelial dysfunction. Insufficient UA may fail to protect against oxidative damage, leading to reduced bone mineral density and impaired healing (12, 14).

Given the potential dual association of UA with hip arthroplasty outcomes, this study hypothesizes that both hypo- and hyperuricemia are independently associated with poorer functional recovery and survival in patients undergoing hip arthroplasty. Understanding this relationship could pave the way for targeted interventions, such as monitoring and modulating UA levels, to improve postoperative outcomes and enhance patient quality of life. Furthermore, this research aims to address a critical gap in the literature by providing robust evidence on the role of UA in hip arthroplasty recovery, potentially informing personalized treatment strategies for at-risk patients.

## Methods

### Study design

**Figure 1:**
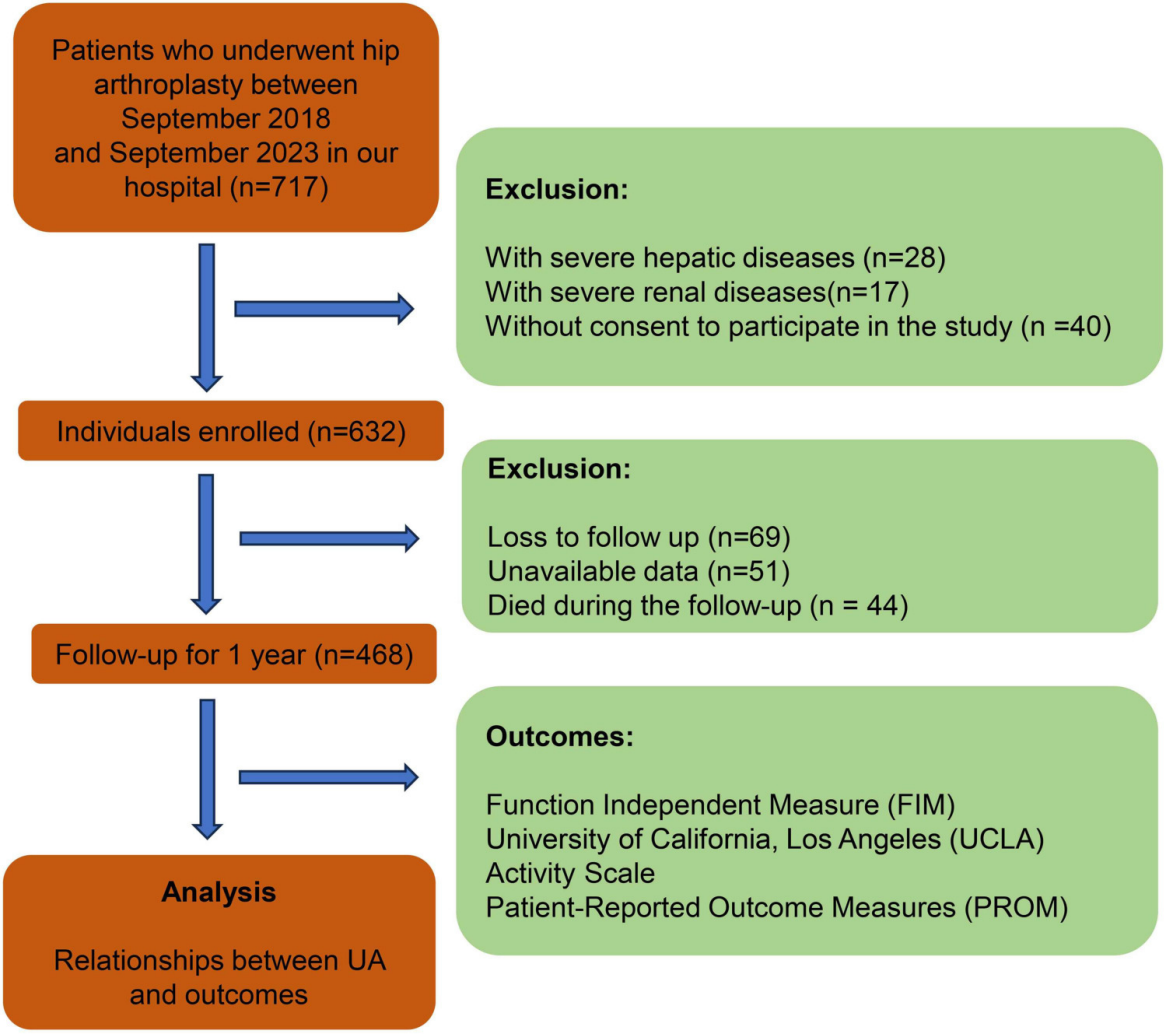
Flow chart of our study.

This study is an observational study conducted at the Department of Orthopedics, the First Hospital of Jilin University. The study was approved by the Ethics Committee of First Hospital of Jilin University (2022-085) and followed the principles of the Declaration of Helsinki. All participants provided written informed consent, and patient privacy was strictly safeguarded. The study included patients who underwent hip arthroplasty in our department between September 2018 and September 2023. The patients who met the specified inclusion criteria were included, and those who met the exclusion criteria were excluded. Inclusion criteria: a. underwent hip arthroplasty (total hip arthroplasty or hemiarthroplasty); b. consent to participate in the study. Exclusion criteria: a. with severe hepatic diseases; b. with severe renal diseases; c. loss to follow-up; d. unavailable data; e. died during the follow-up (Figure 1). All surgeries were performed by the senior author (Dongsong Li) or in his presence and direction.

### Variables

Baseline demographics (age, BMI, sex, residence, smoke and alcoholism history), comorbidities (osteoporosis, hypertension, and so on), fracture history, surgical variables (diagnosis, surgical procedures, and anesthesia), and laboratory parameters including hemoglobin (Hb) (g/L), international normalized ratio (INR), blood glucose (GLU) (mmol/L), albumin (ALB) (g/L) and UA (µmol/L), were extracted from electronic medical records. Abnormal electrocardiograms or radiographs were adjudicated by two independent specialists. Charlson comorbidity index (CCI) was calculated to comprehensively conclude the preoperative comorbid condition (15). Hyperuricemia was defined as UA >420 μmol/L for both males and females according to clinical guidelines.

### Outcomes and follow-up

Patients were prospectively followed for one year. The primary outcome was functional independence, defined as a Functional Independence Measure (FIM) score ≥108 at 1-year follow-up (16). The FIM is a therapist-administered 18-item scale assessing self-care, mobility, and cognition, with total scores ranging from 18 (complete dependence) to 126 (complete independence). Secondary outcomes included: Daily activity level measured by the UCLA Activity Scale (UCLAAS); Patient-Reported Satisfaction (PRS) using Likert-scale questionnaires (1 = "Very dissatisfied” to 5 = "Very satisfied”): Surgery Satisfaction Score (SSS); Pain Satisfaction Score (PSS); Activity Satisfaction Score (ASS); Health Satisfaction Score (HSS); Life Satisfaction Score (LSS).

### Statistical analyses

Continuous variables were presented as mean ± standard deviation, whereas categorical variables were reported as count (percentage). The distribution of continuous variables was evaluated using the Shapiro–Wilk test. Group comparisons utilized the Student's t-test (normal) or the Wilcoxon rank-sum test (non-normal). Categorical variables were analyzed via chi-square/Fisher's exact tests. Restricted cubic splines (RCS) with four knots analyzed nonlinear relationships between UA levels and outcomes based on multiple regression models, and the cutoff values of UA were identified according to the RCS (4). The outcomes of patients with different UA groups were compared. Lastly, multivariable logistic and linear regression (adjusted for age, BMI, and CCI ≥4) quantified UA's association with outcome. The significance level was set at *p* < 0.05, and Bonferroni correction was used to adjust the *p*-values to reduce the impact of multiple tests. All analyses were performed using R software version 4.2.2 (R Foundation for Statistical Computing, Vienna, Austria).

## Results

### Population characteristics

Finally, this study enrolled 468 patients following hip arthroplasty, stratified into functional independence (*n* = 393, FIM ≥108) and non-independence (*n* = 75) groups at 1-year follow-up. The baseline characteristics of patients included in this study are summarized in Table 1. Significant differences were observed in age and comorbidity burden between groups. Patients with functional independence were younger (58.6 ± 12.9 vs. 63.7 ± 13.2 years, *p* < 0.001) and had lower rates of Charlson Comorbidity Index ≥3 (8.65% vs. 18.67%, *p* = 0.009) compared to the non-independence group. No significant differences were noted in sex (54.5% vs. 56.0% female, *p* = 0.289), BMI (24.2 ± 3.9 vs. 23.8 ± 3.9 kg/m^2^, *p* = 0.805), or lifestyle factors (smoking: 44.0% vs. 50.7%, *p* = 0.728). Laboratory parameters revealed higher hemoglobin levels (132.2 ± 19.4 vs. 124.7 ± 21.5 g/L, *p* = 0.003) and lower INR (0.96 ± 0.08 vs. 1.00 ± 0.09, *p* < 0.001) in the independence group 1. Continuous UA levels did not differ significantly between groups (336.4 ± 100.95 vs. 353.63 ± 159.31 μmol/L, *p* = 0.852) while the patients with independence have significantly high rates of HUA (19.34% vs. 32.00%, *p* = 0.014).

**Table 1:** Baseline characteristics of our study.

Variables	All *n* = 468	With independence *n* = 393	Without independence *n* = 75	*p*
Age (years)	59.41 ± 13.07	58.59 ± 12.89	63.72 ± 13.20	0.001
BMI (kg/m^2^)	24.12 ± 3.91	24.17 ± 3.90	23.83 ± 3.95	0.581
Sex (female)	256 (54.70%)	214 (54.45%)	42 (56.00%)	0.805
Residence (rural)	211 (45.09%)	173 (44.02%)	38 (50.67%)	0.289
Smoking history (yes)	19 (4.06%)	17 (4.33%)	2 (2.67%)	0.728
Alcoholism history (yes)	28 (5.98%)	26 (6.62%)	2 (2.67%)	0.291
Osteoporosis (yes)	22 (4.70%)	15 (3.82%)	7 (9.33%)	0.077
Fracture history (yes)	58 (12.39%)	49 (12.47%)	9 (12.00%)	0.91
Hypertension (yes)	142 (30.34%)	116 (29.52%)	26 (34.67%)	0.374
CCI (≥3)	48 (10.26%)	34 (8.65%)	14 (18.67%)	0.009
Electrocardiogram (abnormal)	142 (30.34%)	114 (29.01%)	28 (37.33%)	0.151
Reason for surgery (fracture)	110 (23.50%)	89 (22.65%)	21 (28.00%)	0.316
Surgical procedures (total arthroplasty)	437 (93.38%)	368 (93.64%)	69 (92.00%)	0.787
Surgery side (right)	195 (41.67%)	165 (41.98%)	30 (40.00%)	0.749
Anesthesia (spinal)	93 (19.87%)	77 (19.59%)	16 (21.33%)	0.729
HUA (yes)	100 (21.37%)	76 (19.34%)	24 (32.00%)	0.014
INR	0.97 ± 0.08	0.96 ± 0.08	1.00 ± 0.09	<0.001
Hb (g/L)	131.01 ± 19.93	132.22 ± 19.42	124.69 ± 21.47	0.003
GLU (mmol/L)	5.67 ± 2.31	5.66 ± 2.41	5.76 ± 1.70	0.359
ALB (g/L)	38.33 ± 4.82	38.41 ± 4.54	37.94 ± 6.11	0.127
UA (µmol/L)	339.14 ± 112.32	336.38 ± 100.95	353.63 ± 159.31	0.852

Continuous variables were expressed as mean ± standard deviation, and categorical variables were presented as count (percent). BMI, body mass index; CCI, charlson comorbidity index; INR, international normalized ratio; Hb, hemoglobin; GLU, blood glucose; UA, uric acid; HUA, hyperuricemia.

### Nonlinear associations

**Figure 2:**
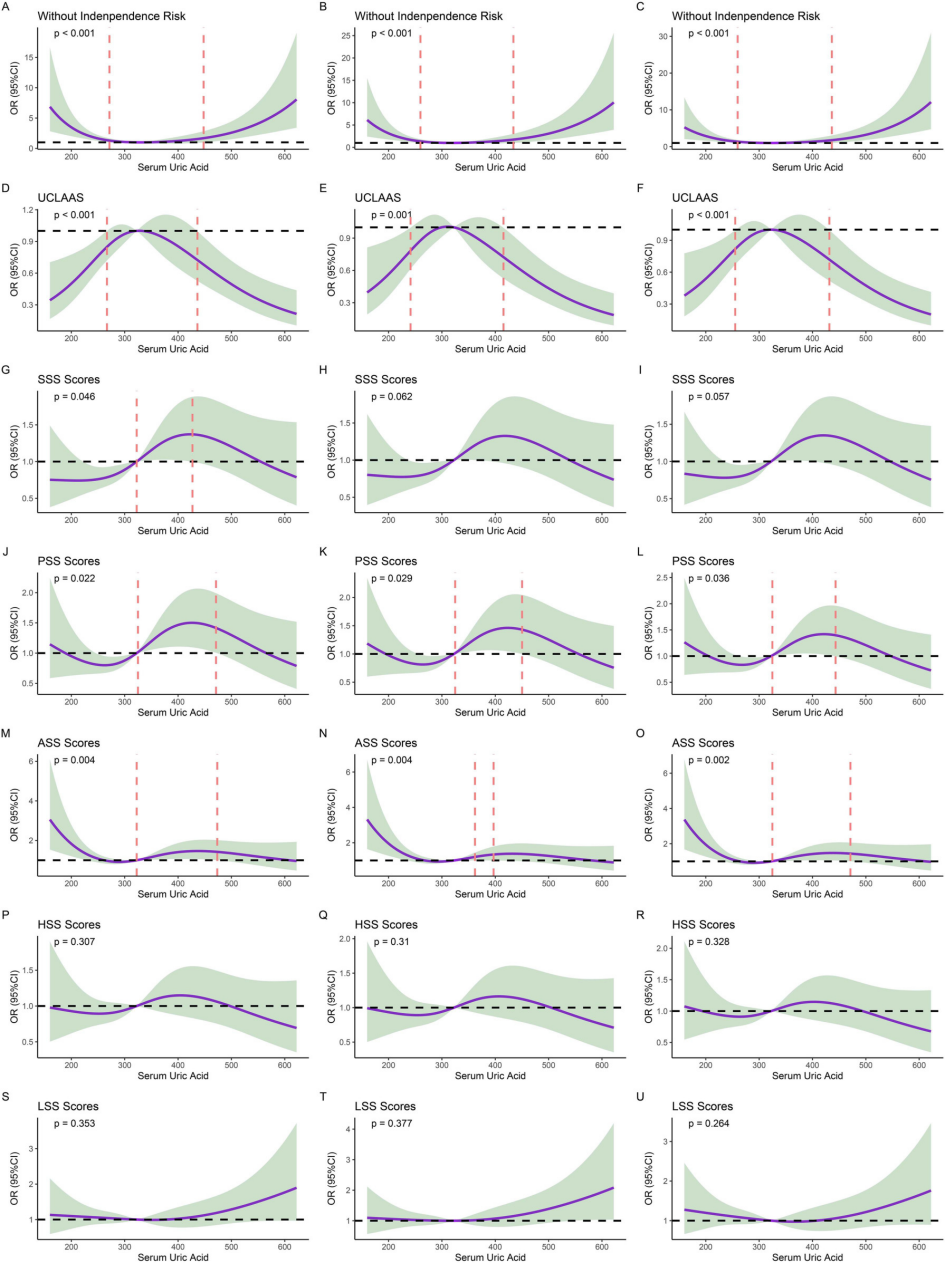
Results of restricted cubic splines (RCS) models. **(A)** non-adjusted models of UA for functional independence; **(B)** models of UA for functional independence and adjusted for age and sex; **(C)** models of UA for functional independence and adjusted for age, CCI, INR, and Hb; **(D)** non-adjusted models of UA for UCLAAS; **(E)** models of UA for UCLAAS and adjusted for age and sex; **(F)** models of UA for UCLAAS and adjusted for age, CCI, INR, and Hb; **(G)** non-adjusted models of UA for SSS; **(H)**: models of UA for SSS and adjusted for age and sex; **(I)** models of UA for SSS and adjusted for age, CCI, INR, and Hb; **(J)** non-adjusted models of UA for PSS; **(K)** models of UA for PSS and adjusted for age and sex; **(L)** models of UA for PSS and adjusted for age, CCI, INR, and Hb; **(M)** non-adjusted models of UA for ASS; **(N)** models of UA for ASS and adjusted for age and sex; **(O)** models of UA for ASS and adjusted for age, CCI, INR, and Hb; **(P)** non-adjusted models of UA for HSS; **(Q)** models of UA for HSS and adjusted for age and sex; **(R)** models of UA for HSS and adjusted for age, CCI, INR, and Hb; **(S)** non-adjusted models of UA for LSS; **(T)** models of UA for LSS and adjusted for age and sex; **(U)** models of UA for LSS and adjusted for age, CCI, INR, and Hb.

RCS analysis revealed a U-shaped relationship between serum uric acid (UA) and functional independence (Figure 2). RCS was established based on three models, including univariate models, models adjusted for age and sex, and models adjusted for the variables with significant differences in Table 1 (age, CCI, INR, and Hb). For the primary outcome, the risk of functional dependence increased at both extremes of UA levels compared to intermediate levels, and this nonlinear pattern persisted across all multivariable models. Similar nonlinear U-shape trends were observed for secondary outcomes in UCLAAS, PSS, and ASS. To better explore the relationships, we further divided the populations according to the cutoff values of UA determined by RCS models: low UA: <259.77 μmol/L; middle UA: ≥259.77 μmol/L and <436.32 μmol/L; high UA: ≥436.32 μmol/L. Moreover, according to the quantiles of UA, the patients were also grouped into Q1–Q4 groups (low values of UA to high levels of UA).

### Outcomes

**Figure 3:**
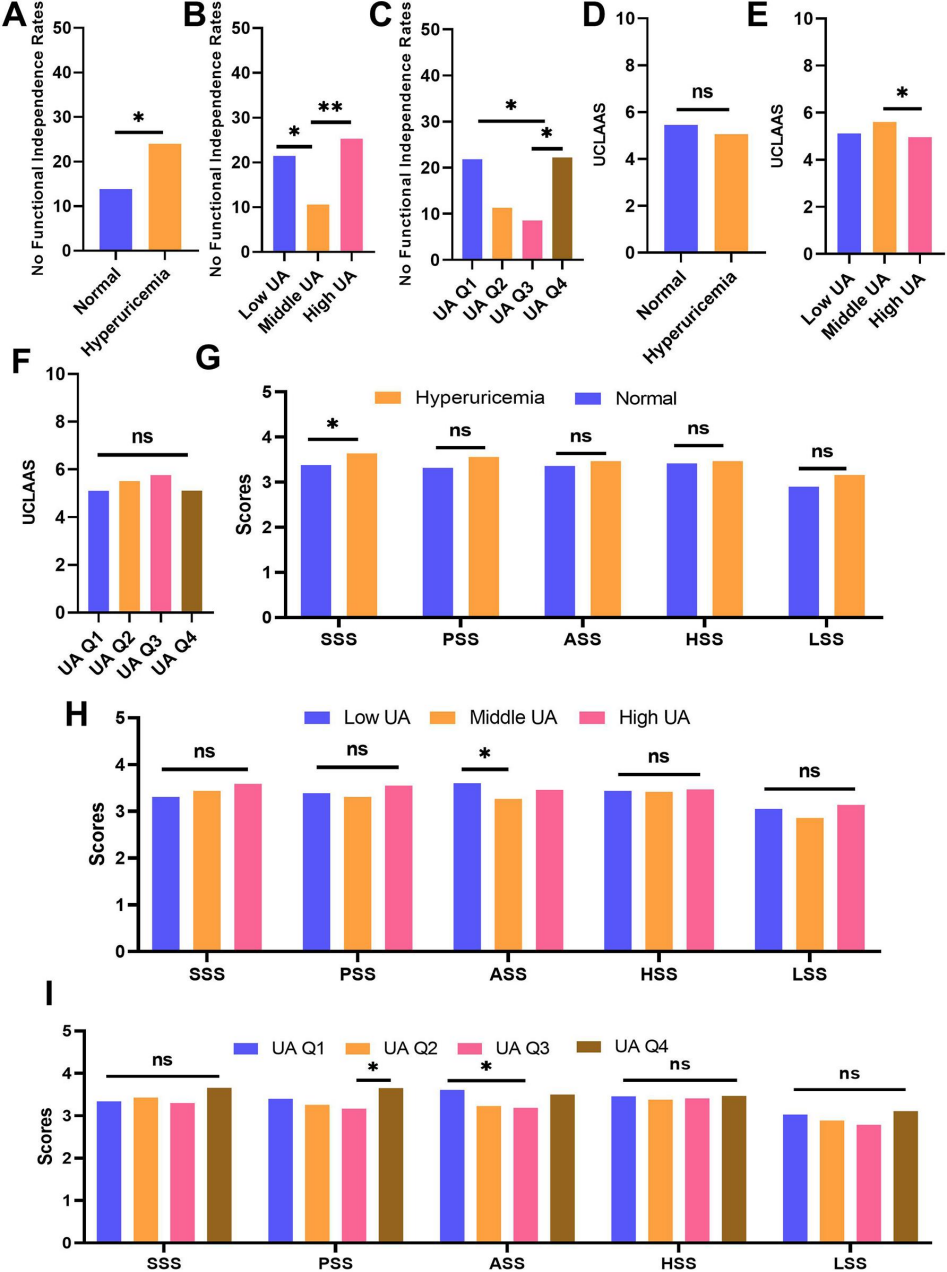
Comparison of outcomes across different UA groups. **(A)** no functional independence rates of individuals grouped by normal and HUA; **(B)** no functional independence rates of individuals grouped by low, middle, and high UA; **(C)** no functional independence rates of individuals grouped by UA quartiles; **(D)** UCLAAS of individuals grouped by normal and HUA; **(E)** UCLAAS of individuals grouped by low, middle, and high UA; **(F)** UCLAAS of individuals grouped by UA quartiles; **(G)** five PRS scores of individuals grouped by normal and HUA; **(H)** five PRS scores of individuals grouped by low, middle, and high UA; **(I)** five PRS scores of individuals grouped by UA quartiles.

Dichotomous (HUA vs. normal), tiered (low/mid/high UA), and quartile-based (Q1-Q4) analyses consistently demonstrated worse functional outcomes at UA extremes (Figure 3). The baseline characteristics of patients grouped by different UA levels were summarized in Supplementary Table S1–S3. HUA was associated with lower functional independence rates and lower SSS scores compared with normal individuals (all *p* < 0.05). Similar to the nonlinear analysis, patients with low UA and high UA may have significantly lower functional independence rates compared with normal individuals. For the secondary outcomes, the adults with high UA may have lower UCLAAS compared with those with middle UA. Surprisingly, patients with moderate UA seemed to have lower ASS scores. In the four-tier system, compared with UA Q3, the patients with UA Q1 and Q4 may have significantly higher nonfunctional independence rates. Similarly, UA Q3 may relate to lower PSS and ASS scores, which seems paradoxical compared with the primary outcome analysis. Sex-stratified analyses confirmed that the U-shaped relationship persisted in both sexes (Supplementary Tables S4, S5). In males, low UA and high UA were linked to higher non-functional independence rates and lower UCLAAS. In females, tiered (low/mid/high UA) and quartile-based (Q1-Q4) UA were associated with PSS and ASS scores, whereas middle UA conferred the most favorable outcomes.

### Multivariate analysis

To avoid the bias caused by co-factors, we further established multivariate models to explore the associations between UA and outcomes. We established three kinds of models for FIM: univariate models, multivariate models adjusted for the factors selected by stepwise method (INR, Hb, age, residence, and reason for surgery), and multivariate models adjusted for factors with significance in univariate models (INR, Hb, age, CCI, and osteoporosis, Supplementary Table S6). The results of multivariable models for FIM were summarized in Table 2. UA as a continuous variable showed no significant association with the primary outcome (OR: 1.001, 95% CI 0.999–1.003; *p* = 0.224) in univariate models, while showing significant associations in multivariate models 1 and 2. HUA and extreme UA levels (Low/High UA, Q1/Q4) exhibited clinically meaningful associations with adverse outcomes (all *p* < 0.05) in all models.

**Table 2:** Logistics models of different UA groups for functional independence.

Variables	Univariate models		Multivariate models 1		Multivariate models 2	
	OR (95% CI)	*p*	OR (95% CI)	*p*	OR (95% CI)	*p*
UA (continuous)	1.001 [0.999, 1.003]	0.224	1.003 [1.000, 1.005]	0.03	1.003 [1.000, 1.005]	0.017
HUA	1.963 [1.124, 3.362]	0.015	2.753 [1.484, 5.076]	0.001	2.954 [1.598, 5.433]	<0.001
Low UA	2.307 [1.281, 4.145]	0.005	2.071 [1.118, 3.833]	0.02	2.092 [1.135, 3.851]	0.017
Middle UA	Ref	Ref	Ref	Ref	Ref	Ref
High UA	2.855 [1.507, 5.357]	0.001	3.464 [1.744, 6.882]	<0.001	3.739 [1.891, 7.407]	<0.001
UA Q1	2.194 [1.082, 4.640]	0.033	2.242 [1.075, 4.875]	0.035	2.269 [1.084, 4.967]	0.034
UA Q2	Ref	Ref	Ref	Ref	Ref	Ref
UA Q3	0.733 [0.301, 1.741]	0.484	0.925 [0.366, 2.290]	0.867	0.945 [0.369, 2.378]	0.905
UA Q4	2.242 [1.105, 4.745]	0.029	3.081 [1.427, 6.979]	0.005	3.288 [1.498, 7.611]	0.004

UA, uric acid; HUA, hyperuricemia. Multivariate models 1 were adjusted by the variables selected by stepwise methods (INR, Hb, age, residence, and reason for surgery), and multivariate models 2 were adjusted by the significantly different variables in univariate models, including INR, Hb, age, CCI, and osteoporosis.

The results of multivariate models for secondary outcomes were summarized in Table 3. For UCLAAS, continuous UA, HUA, high UA, and UA Q4 were all significantly associated with lower UCLAAS scores, while the low UA and UA Q1 were not significant. For other outcomes, low UA and UA Q1 were estimated to relate to higher ASS scores significantly (all *p* < 0.05).

**Table 3:** Multivariate models of different UA groups for secondary outcomes.

Variables	UCLAAS		SSS		PSS	
	Coefficient (95% CI)	*p*	Coefficient (95% CI)	*p*	Coefficient (95% CI)	*p*
UA (continuous)	−0.002 [−0.003, −0.000]	0.035	0.000 [−0.001, 0.001]	0.491	0.000 [−0.001, 0.001]	0.847
HUA	−0.533 [−0.963, −0.102]	0.015	0.205 [−0.078, 0.488]	0.155	0.168 [−0.112, 0.448]	0.238
Low UA	−0.395 [−0.804, 0.013]	0.058	−0.092 [−0.362, 0.178]	0.502	0.121 [−0.145, 0.388]	0.371
Middle UA	Ref	Ref	Ref	Ref	Ref	Ref
High UA	−0.706 [−1.172, −0.240]	0.003	0.115 [−0.193, 0.424]	0.463	0.193 [−0.112, 0.497]	0.214
UA Q1	−0.375 [−0.855, 0.105]	0.126	−0.108 [−0.424, 0.208]	0.5	0.134 [−0.177, 0.445]	0.397
UA Q2	Ref	Ref	Ref	Ref	Ref	Ref
UA Q3	0.155 [−0.341, 0.650]	0.539	−0.215 [−0.541, 0.111]	0.195	−0.172 [−0.493, 0.148]	0.292
UA Q4	−0.512 [−1.017, −0.008]	0.047	0.150 [−0.183, 0.482]	0.377	0.298 [−0.029, 0.625]	0.074
Variables	ASS		HSS		LSS	
	Coefficient (95% CI)	*p*	Coefficient (95% CI)	*p*	Coefficient (95% CI)	*p*
UA (continuous)	−0.001 [−0.002, 0.000]	0.279	−0.000 [−0.001, 0.001]	0.794	0.000 [−0.001, 0.002]	0.632
HUA	0.107 [−0.168, 0.381]	0.446	0.020 [−0.271, 0.310]	0.894	0.169 [−0.175, 0.512]	0.335
Low UA	0.355 [0.095, 0.615]	0.007	0.046 [−0.230, 0.323]	0.742	0.263 [−0.064, 0.590]	0.115
Middle UA	Ref	Ref	Ref	Ref	Ref	Ref
High UA	0.184 [−0.113, 0.481]	0.223	0.037 [−0.279, 0.353]	0.819	0.220 [−0.153, 0.593]	0.247
UA Q1	0.400 [0.096, 0.704]	0.01	0.083 [−0.242, 0.408]	0.617	0.166 [−0.218, 0.550]	0.395
UA Q2	Ref	Ref	Ref	Ref	Ref	Ref
UA Q3	−0.060 [−0.374, 0.253]	0.706	−0.018 [−0.353, 0.318]	0.917	−0.180 [−0.576, 0.217]	0.374
UA Q4	0.265 [−0.055, 0.584]	0.104	0.048 [−0.294, 0.389]	0.784	0.095 [−0.309, 0.498]	0.645

UA, uric acid; HUA, hyperuricemia. Models were adjusted by INR, Hb, age, CCI, and osteoporosis.

## Discussion

This study revealed a U-shaped association between UA and functional outcomes in hip arthroplasty patients, where both low levels of UA and high levels of UA independently predicted reduced functional independence at 1-year follow-up. These findings align with prior evidence demonstrating a dual role of UA in bone health, where extremes disrupt redox balance, exacerbating oxidative stress and impeding recovery. Notably, secondary outcomes exhibited domain-specific effects: while high UA was uniformly detrimental to mobility (UCLAAS: *β* = −0.533, *p* = 0.015), hypouricemia paradoxically correlated with improved activity satisfaction (ASS: *β* = 0.355, *p* = 0.007). This dichotomy highlights context-dependent mechanisms influencing UA's impact on postoperative recovery.

Our results resonate with emerging studies on UA's dual effects on musculoskeletal health. A meta-analysis of 909,803 individuals linked moderate UA levels to higher bone mineral density (BMD) and revealed the U-shape relationships between UA and fracture risk (17). Mechanistically, UA modulates osteoblast-osteoclast equilibrium: low UA may fail to neutralize oxidative stress, impairing bone repair, while high UA may induce inflammation via NLRP3 inflammasome activation and osteocyte apoptosis (18, 19). Hip fracture cohorts similarly report J-shaped mortality curves. Hyperuricemia may amplify endothelial dysfunction and renal impairment, and hypouricemia may exacerbate frailty (4, 20).

Several potential mechanisms of UA may support our conclusion. At physiological levels, UA scavenges reactive oxygen species (ROS), which may potentially protect osteoblasts from oxidative damage (21). At low UA levels, reduced scavenging of reactive oxygen species may accelerate osteoblast apoptosis and impair collagen synthesis, delaying fracture consolidation (22). This is compounded by vitamin D dysregulation—low UA correlates with 25 (OH) vitamin D deficiency, which promotes secondary hyperparathyroidism and bone resorption via upregulated RANKL/OPG signaling (23). Additionally, hypouricemia may exacerbate neuro-muscular dysfunction due to diminished purine metabolite recycling, further hindering rehabilitation efforts (24).

However, on the other side, excess UA generates intracellular ROS and may potentially promote osteoclastogenesis (25, 26). Moreover, HUA accelerates CKD progression, worsening osteoporosis via hyperparathyroidism and phosphate retention—this aligns with our high UA group's increased nonfunction independence risk (27). In the view of inflammation, elevated UA upregulates IL-1β and TNF-α, impairing bone-healing signaling pathways and functional recovery (28).

The inverse association between low UA and improved activity satisfaction (ASS) may reflect behavioral adaptation: patients with lower UA, despite reduced mobility, might engage in compensatory strategies, enhancing perceived satisfaction. Alternatively, hypouricemia-related neuroprotective effects could improve pain tolerance, biasing self-reported scores. Further research is needed to disentangle psychological and organic contributors (29).

Our study has several limitations. First, its single-center design in a tertiary hospital in China may restrict generalizability to other populations and healthcare systems, and the observed U-shaped association should be validated in multicenter, multinational cohorts. In addition to the single-center design, our study carries residual selection bias: the convenience sample of hospitalized patients may have preferentially enrolled individuals in better overall health, potentially underestimating the true risks associated with extreme UA concentrations. Furthermore, detailed information on diet, micronutrient supplementation, and physical activity variables known to influence both uric acid levels and functional recovery was not collected; future multicenter studies should incorporate validated questionnaires and wearable devices to address these unmeasured confounders. Additionally, UA was measured only once during the perioperative period to test its prognostic utility; the absence of serial post-operative UA assessments, together with the lack of bone-turnover and inflammatory biomarkers, such as CTX, P1NP, and IL-6, constrains mechanistic interpretation and should be addressed in future longitudinal studies. Lastly, the paradoxical ASS score findings might also reflect residual confounding from psychosocial factors not captured in clinical metrics.

## Conclusion

Serum UA demonstrates a dual, nonlinear association with functional recovery after hip arthroplasty, where extremes perturb redox balance and bone remodeling. Monitoring perioperative UA levels and targeting individualized thresholds may optimize rehabilitation strategies.

## Data Availability

The raw data supporting the conclusions of this article will be made available by the authors, without undue reservation.

## Supplementary material

Supplementary Table S1

Demographic and clinical characteristics of patients stratified by normal and HUA.

Supplementary Table S2

Demographic and clinical characteristics of patients stratified by low, middle, and high UA.

Supplementary Table S3

Demographic and clinical characteristics of patients stratified by UA quartiles.

Supplementary Table S4

Comparison of outcomes across different UA groups in males.

Supplementary Table S5

Comparison of outcomes across different UA groups in females.

Supplementary Table S6

Results of univariate models of baseline variables.
